# Cannabinoid Ligands and Alcohol Addiction: A Promising Therapeutic Tool or a Humbug?

**DOI:** 10.1007/s12640-015-9555-7

**Published:** 2015-09-09

**Authors:** Patrycja Kleczkowska, Irena Smaga, Małgorzata Filip, Magdalena Bujalska-Zadrozny

**Affiliations:** Department of Pharmacodynamics, Centre for Preclinical Research and Technology (CEPT), Medical University of Warsaw, Banacha 1B, 02-097 Warsaw, Poland; Department of Toxicology, Faculty of Pharmacy, Jagiellonian University, College of Medicum, Medyczna 9, 30-688 Kraków, Poland; Laboratory of Drug Addiction Pharmacology, Department of Pharmacology, Institute of Pharmacology, Polish Academy of Sciences, Smętna 12, 31-343 Kraków, Poland

**Keywords:** Alcohol, Cannabinoids, Co-administration, Endocannabinoid system

## Abstract

The vast therapeutic potential of cannabinoids of both synthetic and plant-derived origins currently makes these compounds the focus of a growing interest. Although cannabinoids are still illicit drugs, their possible clinical usefulness, including treatment of acute or neuropathic pain, have been suggested by several studies. In addition, some observations indicate that cannabinoid receptor antagonists may be useful for the treatment of alcohol dependence and addiction, which is a major health concern worldwide. While the synergism between alcohol and cannabinoid agonists (in various forms) creates undesirable side effects when the two are consumed together, the administration of CB_1_ antagonists leads to a significant reduction in alcohol consumption. Furthermore, cannabinoid antagonists also mitigate alcohol withdrawal symptoms. Herein, we present an overview of studies focusing on the effects of cannabinoid ligands (agonists and antagonists) during acute or chronic consumption of ethanol.

## Introduction

Cannabis is a term used to describe the psychoactive preparations of the plant *Cannabis sativa* that is native to mild to tropical regions of Southeast Asia, the Mediterranean, Central America, and South America. Cannabis is also known as marijuana or ganja, however, this refers to cannabis leaves or other crude plant material, and hashish is a resin extracted from the flower clusters and top leaves of the hemp plant. Each of these products contains a number of biologically active substances of which the most important is delta-9-tetrahydrocannabinol (Δ^9^-THC).

Cannabinoids, both those from cannabis extracts and synthetic preparations, are the most frequently used psychoactive drugs around the world. According to World Health Organization estimates, approximately 147 million people (i.e., 2.5–5 % of the world population) use or abuse cannabis compared with 0.3–0.4 % who take cocaine and 0.3–0.5 % who use opioids (WHO 2012). This high prevalence of cannabis abuse results from the fact that cannabis preparations are not always considered harmful drugs, and thus, are typically admitted to official trading. However, cannabis use is not restricted in every country in the world, for example, marijuana and other cannabis preparations are legal in the Netherlands. The Dutch legislation, where cannabis preparations are considered to be of low social and personal risk associated, voted for a lenient approach and legalization of cannabis distribution. Additionally, the law in the Czech Republic permits to use cannabis for medical purposes, especially in terminally ill people, including AIDS patients and cancer patients treated for chemotherapy-induced nausea and vomiting (Machado Rocha et al. 2008). Furthermore, cannabis has been found to provide relief from spasticity in multiple sclerosis patients (Sastre-Garriga et al. 2011). A variety of applications of cannabis are also observed in United States of America. Despite prohibition by U.S. federal law, approximately 20 states and the District of Columbia allow the use of marijuana for personal, recreational, or medical use. However, the popularity of cannabinoids is still associated with their use as recreational drugs.

Cannabis products, either smoked or taken orally, induce several behavioral effects that may vary depending on the route of administration, emotional state, dose level, and individual vulnerability to certain effects. While euphoria is the most prominent and dominating effect of cannabis use (Ameri 1999), it can also often lead to tachycardia (Lapoint et al. 2011), antinociception (Chiou et al. 2013), as well as memory and cognitive impairment (Abush and Akirav 2012). Several publications showed that use of cannabinoids, especially marijuana, results in a higher (2–6 times) risk of causing road accidents (Masten and Guenzburger 2014). Furthermore, prolonged exposure to plant-derived, synthetic, or endogenous cannabinoid agonists is associated with the development of pharmacological tolerance (Gonzalez et al. 2005).

In spite of so many undesirable side effects exerted by cannabinoids, it is commonly known that these drugs are also used to intensify pleasure in combination with other psychoactive substances, such as alcohol. In fact, it has been observed that heavy cannabis abusers frequently abuse alcohol as well. Co-consumption of these two substances may be highly inadvisable, especially considering that alcohol might compound the undesirable effects of cannabinoids. The effect of alcohol on our brain and body depends on blood alcohol level and duration of drinking. Interestingly, moderate drinking appears to have health benefits, as it may reduce coronary heart disease risk (Baum-Baicker 1985; Kannel and Ellison 1996; Pinder and Sandler 2004). In addition, alcohol can modify people’s emotional state by inducing euphoria, anxiety, or calming effects (Morgan and Badway 2009). However, prolonged regular exposure to alcohol is detrimental to brain cells and may result in serious brain changes associated with brain cell death (Söderpalm et al. 2009). Indeed, like cannabis, chronic alcohol consumption results in detectable impairments in memory (Saults et al. 2007; White 2003) and serious and persistent changes in the brain (e.g., cerebellar degeneration, Marchiafava-Bignami disease, and Wernicke–Korsakoff syndrome) (Ironside et al. 1961; Victor et al. 1971). Additionally, alcohol use can adversely affect the other parts of the body (e.g., liver cirrhosis) (Victor et al. 1959). The development of alcohol tolerance is widely observed in alcohol addicts and can lead to the development of withdrawal symptoms (AWS) when alcohol use is terminated or significantly reduced. Of note, it has been shown that homicide rates rise and fall in accordance with the rise and fall of alcohol consumption (Norstrom 1998; Pridemore 2004).

The above facts suggest that the combination of alcohol and cannabinoids, especially with chronic consumption, may be extremely harmful to the body and mind. However, in contrast to the action of cannabimimetics, several studies have shown that cannabinoid antagonists are efficient in decreasing consumption of alcohol beverages. Thus, combining alcohol consumption with cannabinoid antagonists might provide a new method to treat alcoholism by which the inhibitory action on cannabinoid receptors decreases or blocks alcohol intake by alcohol addicts.

## A Brief History of the Endocannabinoid (eCB) System and Discovery of Its Endogenous Ligands

**Fig. 1:**
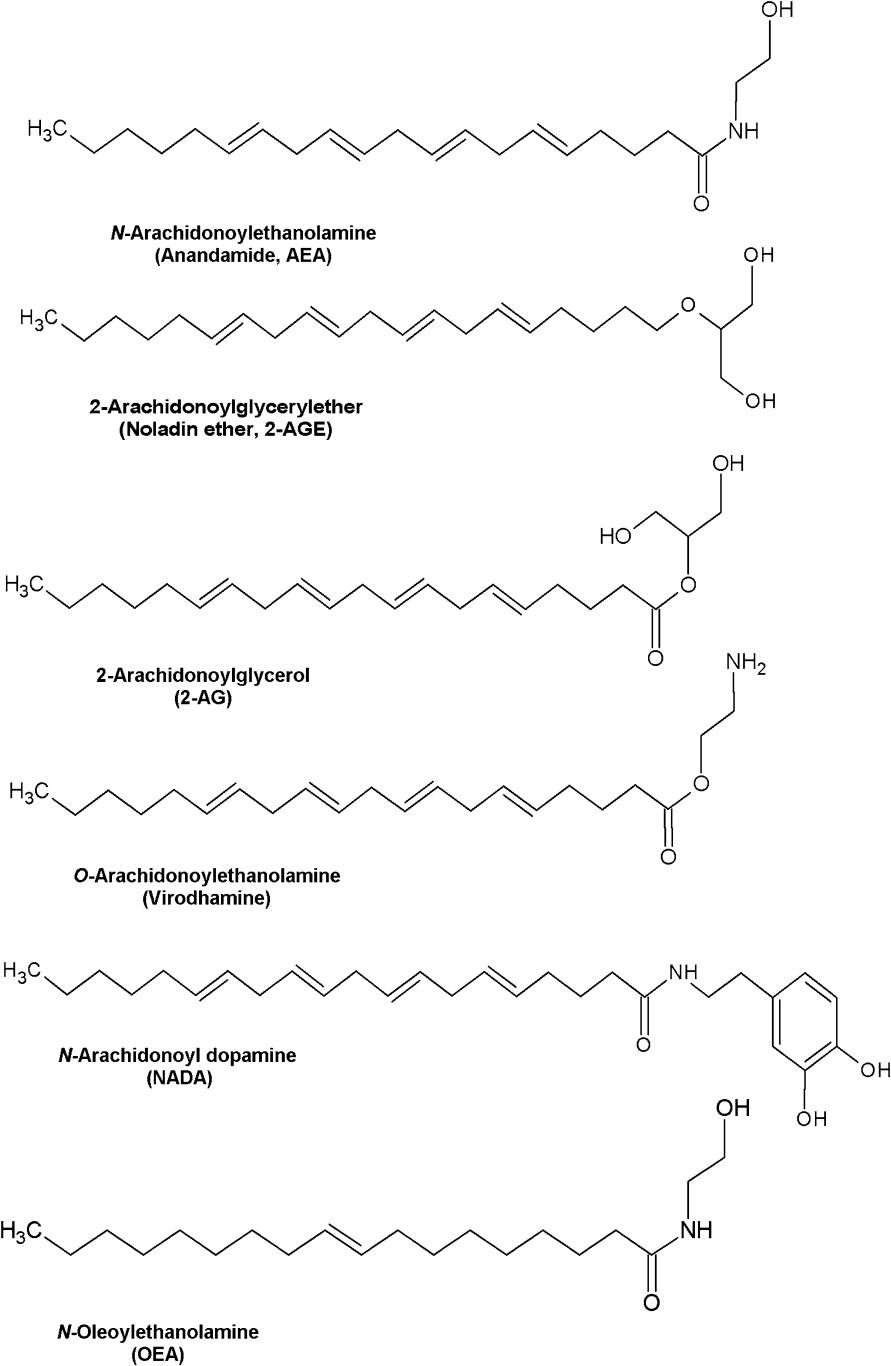
Chemical structures of putative endogenous cannabinoids

Several studies concerning pharmacological effects of cannabinoids in the early 1990s led to the discovery of the endogenous cannabinoid system (Devane et al. 1988, 1992). This unique system consists of two cannabinoid receptors, cannabinoid receptor 1 (CB_1_) and cannabinoid receptor 2 (CB_2_), their endogenous ligands (anandamide, AEA and 2-arachidonoylglycerol, 2-AG) (Fig. 1), and the enzymes that catalyze endocannabinoid (eCB) formation and degradation, such as fatty acid amide hydrolase (FAAH) and monoacylglycerol lipase (Smaga et al. 2014b). The discovery of the first endogenous cannabinoid ligand, which was the ethanol amide of arachidonic acid, took place in 1992. It was named Ananda after a Sanskrit word for pleasure or happiness, and amide due to its chemical structure that distinguishes this endogenous compound from the exogenous agonists (Devane et al. 1992).

In 1995, the second cannabinoid receptor agonist, 2-AG, was discovered (Sugiura et al. 1995). Following this, several other arachidonic acid derivatives were also suggested to be eCBs, including 2-arachidonoylglycerol ether (noladin ether, 2-AGE), *O*-arachidonoyl ethanolamine (virodhamine), *N*-arachidonoyl dopamine (NADA), *N*-arachidonoyl glycine (NAGly), and cis-9,10-octadecanoamide (ODA) (Huang et al. 2001; Leggett et al. 2004; Porter et al. 2002). Unlike other neurotransmitters, AEA and 2-AG are not stored in secretory vesicles but are synthesized “on demand” in the postsynaptic neurons (Ahn et al. 2008).

Most of the actions of eCBs are mediated through the G-protein-coupled receptors, CB_1_ and CB_2_, which are characterized by distinct distributions throughout the body. CB_1_ receptor expression is diffuse and widespread mainly in the periaqueductal gray matter of the brain (Wilson-Poe et al. 2012). Markedly high expression also exists in the substantia nigra, limbic areas (hippocampus, amygdala, cingulate cortex), and the cerebral cortex, especially the frontal cortical areas (Herkenham et al. 1991). Because of their wide distribution on neurons, CB_1_ receptors may play an important role in regulating several physiological processes. For example, CB_1_ receptors are present on axonal terminals, thus having contacts with the GABAergic neurons of the nucleus accumbens (NAc). Consequently, this is found to result in the inhibition of synaptic glutamate release independently of cAMP levels (Rubbie et al. 2001). The presence of CB1 receptors in the dorsal root ganglia is also well known (Morisset et al. 2001), which may contribute to the analgesic effectiveness of cannabinoid agents. Furthermore, co-localization experiments show that CB1 receptors may be present in somatostatin-positive neurons of the lateral septum (Hohmann and Herkenham 2000).

In contrast to CB_1_ receptors, CB_2_ receptors are predominately found in cells of the immune system; however, they are present on both microglia and on neurons in the nervous system (Onaivi et al. 2006). Importantly, although several papers demonstrated CB_2_ receptor expression in almost all of the neurons of a healthy mouse brain, these reports did not include the required negative controls for assessing immunostaining specificity (Gong et al. 2006; Onaivi et al. 2006). Other than these two well-characterized cannabinoid receptors, the existence of a third putative cannabinoid receptor has also been postulated (Ryberg et al. 2007). This orphan G-protein-coupled receptor, known as GPR55, is known to bind to a range of endogenous, plant-derived and synthetic cannabinoid ligands. Despite some similarities with CB_1_ and CB_2_ receptors, the function of this third cannabinoid receptor seems to be far more complex.

### Other Cannabinoid Receptor Ligands (Natural and Synthetic)

**Fig. 2:**
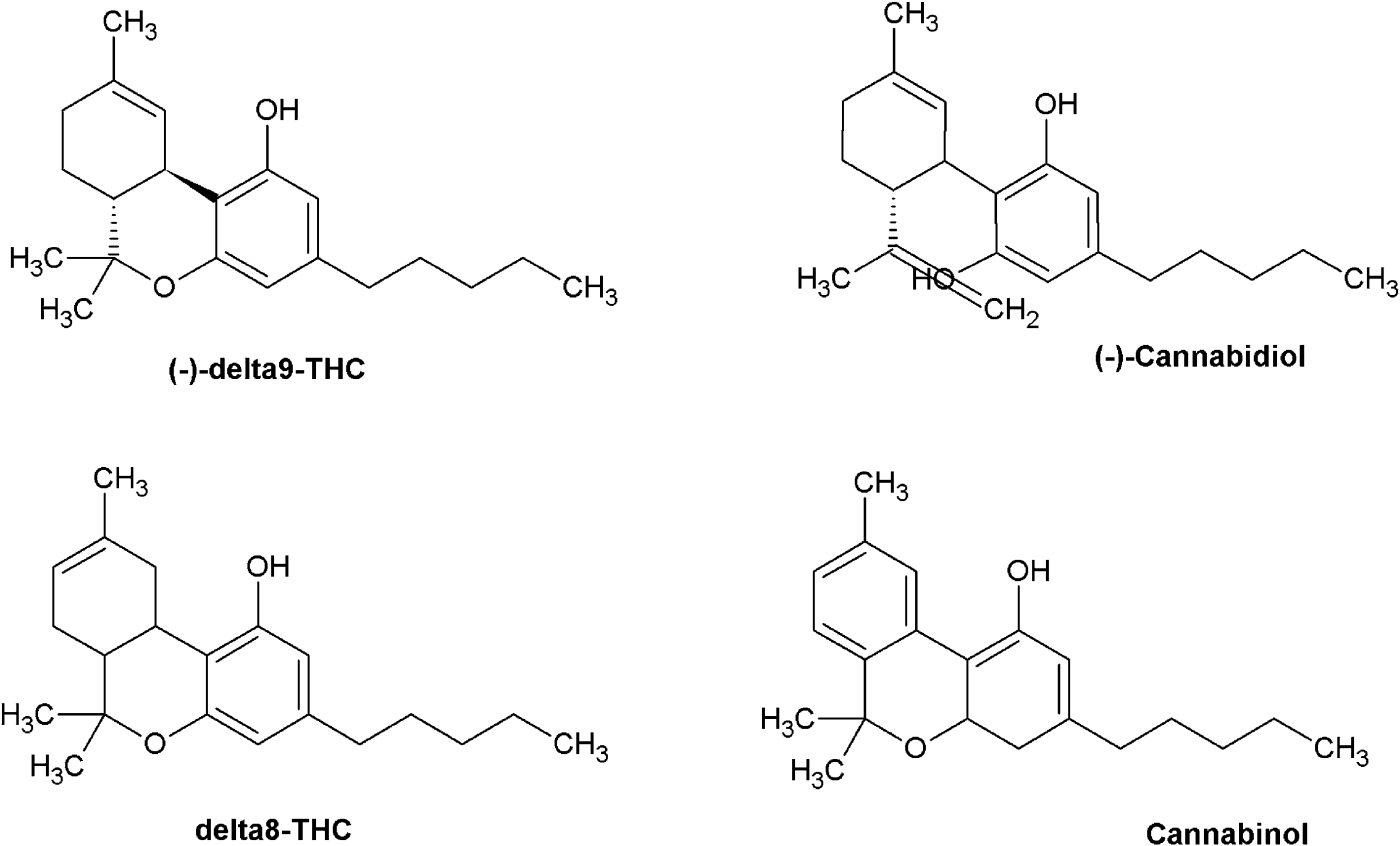
Representative plant-derived cannabinoids

Naturally occurring cannabinoids derived from the cannabis plant constitute a group of chemically related 21-carbon alkaloids, of which Δ^9^-THC is the principal active ingredient, belonging to the herbal cannabinoid family. The activities of Δ^9^-THC and a non-psychoactive cannabinoid of marijuana, cannabidiol (CBD), are well characterized. These compounds may be useful for the treatment of many diseases; CBD has demonstrated neuroprotective and neurogenic effects whereas Δ^9^-THC is widely used as an antinociceptive drug (Consroe et al. 1986; Formukong et al. 1988; Müller-Vahl et al. 1999). Information on the pharmacology and toxicology of the other cannabinoids (Fig. 2) is available, especially regarding their possible roles in treating several diseases (Borrelli et al. 2013) and enhancement of Δ^9^-THC-induced effects (Karniol et al. 1975).

To date, many cannabinoid receptor agonists and antagonists have been synthesized and intensively investigated. Most of them are structurally based on Δ^9^-THC (especially agonists) or mimic its effects. The number of such synthetic agonists has dramatically increased due to the continuously evolving market that is full of synthetic cannabinoid products containing Δ^9^-THC-like ingredients, such as “Spice,” “K2,” “Clockwork Orange,” “Black Mamba” (Zawilska and Wojcieszak 2014). The European Monitoring Centre for Drugs and Drug Addiction (EMCDDA) reported that by March 31st, 2014, almost 110 new synthetic cannabinoids have been detected in “Spice” products available in Europe (Zawilska and Wojcieszak 2014). However, according to the instructions found on the packaging of these products, they are not meant to be consumed by humans, and many users reported smoking or taking such products orally as “speed.”

Due to the continuously increasing list of synthetic cannabinoid compounds and a variety of chemical structures they contain, Howlett et al. (2002) suggested a classification of the synthetic cannabinoids based on their chemical structure. Namely, they described classical, non-classical (HU-308), and hybrid cannabinoid (AM919, AM4030) groups. Additionally, this classification includes aminoalkylindoles (AAIs), which can be further divided into naphthoylindoles (e.g., JWH‐018, JWH‐073, JWH‐398, JWH‐015, JWH‐210, JWH‐081, JWH‐200, WIN‐55,212; AM2201), phenylacetylindoles (e.g., JWH-250, JWH‐251), naphthylmethylindoles (JWH-185, JWH-199), and benzoylindoles (e.g., pravadoline, AM‐694, RSC4);

- eicosanoids (eCBs such as AEA, and their synthetic analogs, e.g., methanandamide);
- and others, including diarylpyrazoles (selective CB_1_ antagonist Rimonabant^®^), naphtoylpyrroles (JWH‐307), naphthylmethylindenes, and derivatives of naphthalene‐1‐yl‐(4‐pentyloxynaphthalen‐1‐yl)methanone (CRA‐13).

The pharmacological properties of some of the presented cannabinoid ligands have been well examined, and many of these compounds appeared to be selective CB_1_ receptor agonists or antagonists. Moreover, some, such as nabilone, have been used for medical purposes, which is currently used for treatment of nausea and vomiting caused by cytotoxic chemotherapy that is unresponsive to conventional antiemetics. More detailed information about the nature of several existing cannabinoid receptor agonists and antagonists is summarized in the next chapters.

#### Agonists

The discovery of Δ^9^-THC and the knowledge of its properties and availability opened a new path to developing highly selective and potent cannabinoid receptor agonists. The intensive synthesis of new compounds, the structures of which are based on the active principal of cannabis, resulted in a great number of active cannabimimetics. Interestingly, several of these novel ligands have a much greater potency than Δ^9^-THC, suggesting that other compounds that are present in herbal marijuana (or other herbal extracts) may influence Δ^9^-THC activity.

Dronabinol is a synthetic Δ^9^-THC formulation and one of the first cannabinoid receptor agonists that displays affinity for CB_1_ and CB_2_ receptors. However, it behaves only as a weak agonist for CB_2_ receptors and is used as an appetite stimulant in HIV patients with anorexia. As shown by Beal et al. (1995), administration of capsules containing 2.5 mg of dronabinol twice a day caused an average weight gain of 0.1 kg versus an average loss of 0.4 kg in the placebo group (Beal et al. 1995). Additionally, several papers have demonstrated that this novel compound is an efficient analgesic and is a useful therapy for chronic non-cancer pain patients who take stable doses of opioids (Narang et al. 2008). Finally, agonist substitution has been an effective strategy for promoting abstinence from many substances of abuse (e.g., nicotine, opioids), and dronabinol has been proposed to serve as a replacement drug in cannabis addiction (Vandrey et al. 2013), especially in cases where withdrawal can be a significant barrier to cessation. Like nabilone it is used as an antiemetic drug in vomiting induced by cytotoxic agents (Tramèr et al. 2001).

Nabilone (Cesamet^®^) is another synthetic cannabinoid characterized by an affinity of 2.2 nM for human CB_1_ and 1.8 nM for CB_2_ receptors. This compound is used to treat nausea associated with cancer chemotherapy (Maida et al. 2008). Nabilone also appears to be a promising candidate agonist for substitutive medication for cannabinoid agonist replacement treatment of cannabis use disorders (Lile et al. 2010). In addition, consumption of nabilone resulted in drug-appropriate responses in subjects who learned to discriminate from Δ^9^-THC (Lile et al. 2010). Several other studies have indicated that nabilone is a potent and effective drug for the treatment of various disorders, mainly related to pain. For example, in a randomized, double-blind placebo-controlled trial, nabilone suppressed pain and increased functional capacity in fibromyalgia patients (Skrabek et al. 2008).

Similar analgesic properties are also observed with Sativex, a cannabinoid extract oral spray containing both Δ^9^-THC and CBD, which is commercially available for neuropathic pain and spasticity in 25 countries primarily in Europe (Myers and Shetty 2008; Nurmikko et al. 2007; Palmer 2014). A selective CB_2_ cannabinoid receptor agonist, O-3223, is an additional compound that can reduce pain and inflammation without apparent cannabinoid-like behavioral effects (Kinsey et al. 2011).

Considering the pharmacological profile of other cannabinoid ligands, WIN 55,212-2 appears to be very interesting. Aside from its antinociceptive activity, this nanomolar affinity cannabinoid receptor agonist (K_i_ values of 62.3 and 3.3 nM at the human recombinant CB_1_ and CB_2_ receptors, respectively) has also been reported to reduce endothelial cell (EC) inflammatory responses induced by bacterial lipopeptide and TNFα as well as to promote neural repair processes after neonatal hypoxia–ischemia (Fernandez-Lopez et al. 2010; Wilhelmsen et al. 2014). Importantly, such a neuroprotective activity was also observed in both global and focal cerebral ischemia and was blocked by a selective CB_1_ receptor antagonist, SR141716A (Nagayama et al. 1999; Shen et al. 1996). Unfortunately, this antagonist also caused several side effects and enhanced locomotor responses when combined with other psychoactive drugs (e.g., heroin, amphetamine) (Lamarque et al. 2001). On the contrary, CP-55,940, another cannabimimetic, did not enhance sensitivity to the behavioral effects of cocaine. Moreover, its co-administration with cocaine reduced the locomotor hyperactivity produced by the psycho-stimulant (Arnold et al. 1998). Despite the fact that this compound is found to be almost 45 times more potent than Δ^9^-THC and is considered to be a full agonist at both CB_1_ (*K*_*i*_ = 0.58 nM) and CB_2_ (*K*_*i*_ = 0.68 nM) receptors, it has not yet been approved for human medical use, as the adverse effects and long-term damage and addiction potential of CP-55,940 are not yet known.

The list of potent agonists at either CB_1_ or CB_2_ receptors is relatively long. Despite their reported efficacy in many biological conditions, most cannabinoid agonists are not currently used in clinic and are instead used as tools for cannabinoid research.

#### Antagonists

SR141716A, also known as rimonabant, is the most potent and orally active antagonist of the CB_1_ receptor. This compound, discovered by the laboratory of Rinaldi-Carmona, displays nanomolar affinity for CB_1_ (*K*_*i*_ = 1.98 nM) and micromolar for human CB_2_ receptors expressed in CHO cells (Rinaldi-Carmona et al. 1994). Intensive studies have suggested its anorectic activity (Freedland et al. 2000). Indeed, several papers demonstrated that intake of different diets seemed to be sensitive to dose-dependent, antagonist-induced cannabinoid receptor blockade, which does not significantly alter other normal behaviors (Freedland et al. 2000; Tucci et al. 2006). Accordingly, other well-known antagonists are able to exert similar effects as rimonabant, including AM251 [*N*-(piperidin-1-yl)-5-(4-iodophenyl)-1-(2,4-dichlorophenyl)-4-methyl-1H-pyrazole-3-carboxamide], which is a selective and potent CB_1_ receptor antagonist with well-characterized anorectic effects in freely fed animals (Hildebrandt et al. 2003); and novel antagonists LH21 (Pavon et al. 2006), and URB477 (LoVerme et al. 2009).

Due to the anti-obesity activity of CB_1_ receptor antagonists, they may be considered to be promising tools to treat obesity disorders. However, application of these compounds leads to several serious adverse effects. For instance, the study by Moreira and Crippa (2009) showed that CB_1_ receptor blockade impaired fear elimination (Moreira and Wotjak 2010). Additionally, an increased incidence of depression and aggression has been reported, including an increase in suicidal thoughts (Christensen et al. 2007).

On the contrary, the first CB_2_ receptor antagonist SR144528, having *K*_*i*_ values of 0.6 nM at CB_2_ and 400 nM at CB_1_ (Rinaldi-Carmona et al. 1998), as well as other recently discovered CB_2_ receptor antagonists (e.g., JTE-907) can markedly reduce ear swelling in chronic contact dermatitis induced either by repeated challenge with oxazolone (Oka et al. 2006) or by repeated exposure to dinitrofluorobenzene in mice (Ueda et al. 2005). However, in comparison with some of the CB_1_ receptor agonists and antagonists, none of the existing CB_2_ receptor antagonists are listed as controlled substances worldwide.

Of importance, because many CB_1_ antagonists are, in fact, partial or inverse agonists (e.g., AM281, AM251, SR141716A), they may produce significantly different effects via different mechanisms of action. These effects may also depend on the state of endocannabinoid signaling pathway. Therefore, further studies are warranted to establish the complete pharmacological profile of such compounds.

## The Endocannabinoid System and Alcohol

The eCB system is involved in the modulation of emotional responses, memory, learning, and reward systems. Importantly, there are many indications that this system is involved in alcohol-induced impairments, namely CB_1_ receptor function is implicated in functional modulation of the mesocorticolimbic dopaminergic pathway and motivation for drug seeking (Bystrowska et al. 2014; Parolaro et al. 2007; Smaga et al. 2014a, b). Several biochemical studies revealed changes within the components of the eCB system after acute and chronic alcohol intake (Basavarajappa and Hungund Basavarajappa and Hungund 1999a, 1999b; Basavarajappa et al. Basavarajappa et al. 2000a, 2000b; Gonzáles et al. Gonzalez et al. 2002a, b and Gonzalez et al. 2002c). Pharmacological studies using CB ligands found alterations in alcohol-related behavior while genetic studies demonstrated changes in different rodent strains (Erdozain and Callado 2011).

### The effects of Ethanol Exposure on the eCB System

#### Preclinical Studies

Preclinical experiments demonstrated that acute exposure to alcohol reduced tissue concentrations of AEA in several rat brain structures including limbic and subcortical areas (Ferrer et al. 2007; Rubio et al. 2007) and the level of 2-AG in the prefrontal cortex (Rubio et al. 2007) (see Table 1). Interestingly, the reduction in eCB levels following acute alcohol administration was correlated with a decrease in glutamate release, which can modulate the release of other neurotransmitters (Ferrer et al. 2007). In contrast with tissue levels, eCBs levels increased in dialysates and in hippocampal cell cultures, (Basavarajappa et al. 2008) whereas 2-AG levels increased in the nucleus accumbens (Caille et al. 2007) after acute alcohol treatment. The increased 2-AG levels in limbic structures may be involved in the elevated alcohol consumption and preference in methamphetamine-lesioned mice that serve as a model of enhanced alcohol intake (Gutierrez-Lopez et al. 2010). Fatty acid amide hydrolase (FAAH) activity was reduced in the hypothalamus, prefrontal cortex (Rubio et al. 2009), and hippocampus (Ferrer et al. 2007) after acute administration of alcohol. However, the levels of FAAH protein were increased, probably as a compensatory response to the primary effect of alcohol on its enzyme function (Rubio et al. 2009). In line with the reduced FAAH activity and enhanced AEA levels, CB_1_ receptor density decreased in the prefrontal cortex and amygdala (Rubio et al. 2009) (Table 1).

**Table 1:** Modulation of the eCB system by alcohol (acute, chronic) and its withdrawal

Alcohol (dosage, route, treatment)	Animal	Analysis	eCB system change				References
			eCBs levels		Degradative enzyme (faah)	eCB_1_ receptor density	
			AEA	2-AG			
Acute exposure							
10 % (w/v) EtOH, self-administration, 30 min	Wistar rat	Dialysate	ϕ nucleus accumbens	↑ nucleus accumbens	n.d.	n.d.	Caille et al. 2007
8 % (v/v) EtOH in liquid diet, 24 h access	Sprague rat	Tissue	↓ hypothalamus ↓ amygdala ↓ caudate-putamen ϕ prefrontal cortex	ϕ hypothalamus ϕ amygdala ϕ caudate-putamen ↓ prefrontal cortex	n.d.	n.d.	Rubio et al. 2007
4 g/kg, i.p., acute	Wistar rat	Tissue	↓ cerebellum ↓ hippocampus ↓ nucleus accumbens	n.d.	ϕ cerebellum ↓ (activity) ϕ (protein) hippocampus ϕ nucleus accumbens	mRNA: ϕ cerebellum ϕ hippocampus	Ferrer et al. 2007
8 % (v/v) EtOH in liquid diet, 24 h access	Sprague rat	Tissue	n.d.	n.d.	↓ (activity) ↑ (protein) hypothalamus ϕ amygdala ϕ caudate-putamen ↓ (activity) ϕ (protein) prefrontal cortex	ϕ hypothalamus ↓ amygdala ϕ caudate-putamen ↓ prefrontal cortex	Rubio et al. 2009
50 mM EtOH, 30 min application	C57BL6/J hippocampal cell culture	Cell extract	↑ hippocampus	↑ hippocampus	n.d.	ϕ hippocampus	Basavarajappa et al. 2008
Chronic exposure							
Chronic intermittent forced vapor inhalation, 49 days	Wistar rat	Tissue	n.d.	n.d.	n.d.	mRNA: ϕ cortex	Rimondini et al. 2002
7.2 % (v/v) EtOH, liquid diet, 15 days	Wistar rat	Tissue	ϕ cerebellum ϕ brainstem ↓ midbrain ϕ hippocampus ↑ limbic forebrain ϕ striatum ϕ diencephalon ϕ cerebral cortex	ϕ cerebellum ϕ brainstem ↓ midbrain ϕ hippocampus ϕ limbic forebrain ϕ striatum ϕ diencephalon ϕ cerebral cortex	n.d.	n.d.	Gonzalez et al. 2002a
7.2 % (v/v) EtOH, liquid diet, 15 days	Wistar rat	Tissue	n.d.	n.d.	n.d.	ϕ cerebral cortex ϕ cerebellum ϕ caudate-putamen ϕ ventromedial hypothalamic nucleus ϕ basolateral amygdala ϕ septum nuclei hippocampal areas: ϕ CA1 ϕ CA2 ϕ CA3 ϕ dentate gyrus	Gonzalez et al. 2002b
Forced consumption of 10 % (v/v) EtOH, 52 days	Wistar rat	Tissue	n.d.	n.d.	n.d.	↓ caudate-putamen- hippocampal areas: ↓ CA1 ↓ CA2 ϕ CA3 ↓ dentate gyrus ↓ ventromedial nucleus of the hypothalamus	Ortiz et al. 2004
Operant alcohol self-administration, from 4.93 (1 day) to 8.36 (18 day) g/kg; 18 days	Marchigian Sardinian alcohol-preferring (msP) rats	Tissue	n.d.	n.d.	n.d.	mRNA: ϕ frontal cortex ϕ cingulate cortex ϕ frontoparietal cortex ↓ caudate-putamen ϕ central amygdaloid nucleus ϕ basolateral amygdaloid nucleus ϕ dorsal hippocampal subregions ↓ pituitary	Cippitelli et al. 2005
Forced vapor inhalation, 72-96 h	Swiss Webster mice	Synaptic plasma membrane	n.d.	n.d.	n.d.	↓ whole brain	Basavarajappa et al. 1998a, b; Basavarajappa and Hungund 1999b
Forced vapor inhalation, 72 h	Swiss Webster mice	Tissue	↑ cortex	n.d.	↓ cortex	↓ cortex ↓ hippocampus ↓ striatum ↓ cerebellum	Vinod et al. 2006
Twice daily, 2 or 4 g/kg; i.p.; 10 days	C57Bl/6 J mice	Tissue	n.d.	n.d.	n.d.	protein: ϕ lateral cortex ϕ medial cortex ϕ dorsal hippocampus ϕ nucleus accumbens ↑ ventral tegmental area ↓ hypothalamus ϕ periaqueductal gray ϕ cerebellum	Pava et al. 2012
50–150 mM, 24–72 h, chronic,	neuroblastoma SK-N-SH cells	Cell extract	n.d	n.d.	n.d.	n.d.	Basavarajappa and Hungund 1999a
100–150 mM, 48–72 h, chronic,	cerebellar granule neurons	Cell extract	n.d.	↑ cerebellum	n.d.	n.d.	Basavarajappa et al. 2000a, b
100–150 mM, 72 h, chronic,	cerebellar granule neurons	Cell extract	↑ cerebellum	n.d.	↓ (activity) cerebellum	n.d.	Basavarajappa et al. 2003
Withdrawal							
Chronic intermittent forced vapor inhalation, 49 days + 3 week withdrawal	Wistar rat	Tissue	n.d.	n.d.	n.d.	mRNA: ↑ cortex	Rimondini et al. 2002
7.2 % (v/v) EtOH, liquid diet, 10 days + 3 h withdrawal	Wistar rat	Tissue	ϕ hypothalamus ↓ amygdala ↓ caudate-putamen	ϕ hypothalamus ϕ amygdala ϕ caudate-putamen	n.d.	ϕ cerebral cortex ϕ caudate-putamen ↓ globus pallidus ↓ substantia nigra ϕ amygdala	Rubio et al. 2008
Oral chronic intermittent (60 doses-5 doses-5 g/kg + 55 doses-6 g/kg) + 2-40 day withdrawal	Sprague rat	Tissue	hippocampus-(2 days) hippocampus ↑ (40 days)	hippocampus ↑ (2 days) hippocampus↑ (40 days)	n.d.	protein, mRNA: ↓ (2 days) hippocampus ↑ (40 days) hippocampus-	Mitrirattanakul et al. 2007
Forced vapor inhalation, 72 h + 24 h withdrawal	Swiss Webster mice	Tissue	Cortex-reversed	n.d.	n.d.	cortex-reversed ↑ hippocampus striatum-reversed cerebellum-reversed	Vinod et al. 2006
Twice daily, 2 or 4 g/kg; i.p.; 10 days + 10 day withdrawal	C57Bl/6 J mice	Tissue	n.d.	n.d.	n.d.	Protein: ϕ lateral cortex ϕ medial cortex ϕ dorsal hippocampus ϕ nucleus accumbens ϕ ventral tegmental area ϕ hypothalamus ϕ periqueductal gray ϕ cerebellum	Pava et al. 2012

*EtOH* ethanol, *n.d* no data, ↑ increase, ↓—decrease, ϕ—none effect

Chronic alcohol intoxication evoked a reduction in eCB levels in the midbrain and an increase in AEA levels in the limbic forebrain in rats (Gonzalez et al. 2002a). The increased AEA levels in the limbic area, which is a key region for drug reinforcement processes, may be involved in the eCB-induced elevation of synaptic transmission, which leads to the addictive properties of alcohol. At the same time, reduced eCB levels in the midbrain may be a compensatory response induced by the activation of the negative feedback regulatory loop from the limbic forebrain to the ventral tegmental area (Gonzalez et al. 2002a). In another study, AEA levels increased in the cortex in mice simultaneously with decreasing cortical FAAH activity (Vinod et al. 2006). The rise in AEA levels may be associated with the activation of phospholipase A_2_ (PLA_2_), which is a crucial enzyme for eCB formation as shown in an animal model of chronic alcohol consumption (Basavarajappa et al. 1998b). It was also documented that chronic alcohol exposure might increase the levels of eCBs by inhibiting the AEA transporter in neurons exposed to chronic alcohol, and chronic alcohol additionally prevented AEA inactivation (Basavarajappa et al. 2003).

It was speculated that a rise in the levels of eCBs led to down-regulation of the CB_1_ receptors. In fact, chronic exposure to alcohol evoked a reduction in CB_1_ receptor density in mice (Basavarajappa et al. 1998a; Basavarajappa and Hungund 1999b; Vinod et al. 2006) and in rats (Ortiz et al. 2004). However, this phenomenon was not observed in earlier research in the rat brain (Gonzalez et al. 2002b; Rimondini et al. 2002) likely due to differences between the two experimental designs (alcohol intake, experimental time, etc.).

Moreover, chronic alcohol consumption may inhibit some processes related to gene expression. In Marchigian Sardinian alcohol-preferring (msP) rats, CB_1_ receptor mRNA expression was increased and chronic alcohol consumption reduced CB_1_ receptor mRNA levels in the caudate-putamen and pituitary, the brain areas relevant to the processing of reward and reward-associated behaviors (Cippitelli et al. 2005). In C57Bl/6J mice, chronic alcohol consumption induced a decrease in CB_1_ receptor density in the hypothalamus and an increase in CB_1_ density in the ventral tegmental area (Pava et al. 2012). Ten-day treatment of mice with alcohol also resulted in a reduction in the hypolocomotive, antinociceptive, and hypothermic effects of an acute dose of the CB_1_ agonist WIN 55,212-2, while a 10-day withdrawal period reversed this effect (Pava et al. 2012). Together, these data suggest that CB_1_ receptors play an important role in the neurochemical processes related to alcohol consumption.

Additionally, Agudelo et al. (2013) was the first to provide evidence of up-regulation of CB_2_ and GPR55 (the third putative cannabinoid receptor) in monocyte-derived dendritic cells from alcohol abusers. Ethanol-treated cells demonstrated higher levels of CB_2_ and GPR55 mRNA. Furthermore, it was observed that alcohol significantly modulated dendritic cells to produce higher levels of the pro-inflammatory cytokine IL-1β (Agudelo et al. 2013). Thus, these receptors may play an immunoprotective role during alcohol-induced immune dysfunction. It has also been reported that CB_2_ receptor agonists have the ability to positively regulate Kupffer cells, which result in liver injury when activated by endotoxins in alcohol drinking individuals, and CB_2_ receptors play a role in alcohol-induced inflammation (Louvet et al. 2011).

In vitro studies showed increased AEA (Basavarajappa et al. 2003) and 2-AG (Basavarajappa et al. Basavarajappa et al. 2000a, b) levels in cerebellar granule neurons with reduced FAAH activity (Basavarajappa et al. 2003) and increased AEA levels in neuroblastoma SK-N-SH cells after chronic alcohol exposure (Basavarajappa and Hungund 1999a) (Table 1). A rise in these lipid mediators may serve as a neuronal compensatory adaptation in the chronic presence of alcohol, and it should be noted that this increase was not dependent on alcohol-induced AEA transporter inhibition.

Alcohol abstinence also provoked changes within the eCB system. The increase in AEA levels was reversed in the mouse cortex (Vinod et al. 2006) and in the rat amygdala and caudate-putamen (Rubio et al. 2008). The reduced level of AEA was accompanied by a low glutamate concentration in the amygdala, and thus, it appears to be related to reduced NAPE-phospholipase D activity that depends on glutamate-mediated calcium influx (Hansen et al. 2000; Rubio et al. 2008). Another study indicated that eCB levels increased in the hippocampus after 40 days of abstinence. Specifically, increased 2-AG levels were rapidly observed only after 2 days of abstinence. Interestingly, CB_1_ receptor levels were reduced after 2 days and increased after 40 days of alcohol abstinence (Mitrirattanakul et al. 2007). The increased eCB production was the result of chronic increases in hippocampal excitability on the terminals of GABAergic interneurons and in consequence may have reduced long-term increases in CB_1_ activation on GABA release (Mitrirattanakul et al. 2007). An increase in CB_1_ receptors was also observed in the cortex (Rimondini et al. 2002) and hippocampus (Vinod et al. 2006), while alcohol abstinence reversed the reduced levels of CB_1_ in the cortex, striatum, and cerebellum in rats (Vinod et al. 2006). On the other hand, a reduction of CB_1_ receptors was found in the globus pallidus and substantia nigra (Rubio et al. 2008). In C57Bl/6J mice, 10-day abstinence evoked normalization in CB_1_ protein expression in the hypothalamus and ventral tegmental area (Pava et al. 2012). Apart from the present review, there is another excellent paper showing in details the preclinical interactions between alcohol and the brain eCB system (Pava and Woodward, 2012). Moreover, the effects of drugs of abuse (particularly cocaine) on the eCB system are described in the recent review paper of Vlachou and Panagis (2014). Based on the above preclinical knowledge of the role of the eCB system in processes related especially to alcohol consumption further studies on alcohol use disorder in which the eCB system plays a crucial role are urgently required.

#### Clinical Studies

In *postmortem* studies, elevated levels of eCBs were observed in the dorsolateral prefrontal cortex of alcoholic suicide victims and were suggested to inhibit GABAergic signaling and provoke impulsivity (Vinod et al. 2005). The increased eCBs levels may also play a role in reinforcing the effects of alcohol or may act more directly as a neuroprotective adaptation to chronic alcohol abuse. Although CB_1_ receptors were increased in the dorsolateral prefrontal cortex (Vinod et al. 2005) and in the ventral striatum (Vinod et al. 2010) of alcoholic suicide victims, the latter changes were not associated with alcohol dependence, as a down-regulation of CB_1_ receptors was noted in alcohol-dependent non-suicide victims (Smaga et al. 2014b; Vinod et al. 2010). An increase in FAAH activity was identified in the ventral striatum of alcohol-dependent suicide victims compared with alcohol-dependent non-suicide victims (Vinod et al. 2010). Another study showed a decrease in AEA levels in the nucleus accumbens and frontal cortex of Cloninger type 1 alcoholics with a reduced dopaminergic transmission in the accumbal reward system (Lehtonen et al. 2010).

### The effects of eCB system modulation on alcohol consumption

#### Preclinical Studies

A large body of data suggested that a genetic predisposition to alcohol abuse depends on a disturbance of the eCB system. In fact, CB_1_ receptor knockout mice generated on a CD_1_ genetic background (outbred CD_1_ mouse strain) demonstrated a reduction in alcohol-induced conditioned place preference (CPP) (Houchi et al. 2005; Thanos et al. 2005) and a decrease in consumption of alcoholic solution in the two-bottle choice test (Naassila et al. 2004). The same decreased voluntary intake of alcohol was demonstrated in C57BL/6J mice with genetic deletion of the CB_1_ receptor gene (Lallemand and de Witte 2005; Poncelet et al. 2003; Vinod et al. 2008b; Wang et al. 2003). Additionally, genetic deletion of FAAH in C57BL/6J mice provoked an increase in voluntary alcohol intake in the two-bottle choice protocol (Basavarajappa et al. 2006; Blednov et al. 2007; Vinod et al. 2008a). These studies confirmed the involvement of CB_1_ receptors in alcohol abuse, and pharmacological blockade of these receptors seems to be a rational approach to the treatment of alcohol addiction.

Accumulated experimental data on cannabis and alcohol interactions have suggested that cannabinoids may act either as substitutes in alcohol withdrawal by counteracting withdrawal symptoms such as tremor and nausea, or as therapeutic agents to help in alcohol cessation. Indeed, it has been demonstrated that stimulation of eCB signaling using the cannabinoid receptors agonists CP55,940 (Colombo et al. 2002; Gallate et al. 1999; Vinod et al. 2008b) and WIN 55,212-2 (Alen et al. 2009; Colombo et al. 2002; Linsenbardt and Boehm 2009; Lopez-Moreno et al. 2004), the selective AEA reuptake inhibitor AM404 (Cippitelli et al. 2007) or the FAAH inhibitor URB597 (Blednov et al. 2007; Cippitelli et al. 2008; Hansson et al. 2007; Vinod et al. 2008a) influences alcohol intake. It was reported that activation of the eCB system increased alcohol consumption. Specifically, acute administration of non-selective CB_1_/CB_2_ receptor agonists CP55,940 (Colombo et al. 2002; Gallate et al. 1999; Vinod et al. 2008b) and WIN 55,212-2 (Alen et al. 2009; Colombo et al. 2002; Linsenbardt and Boehm 2009; Lopez-Moreno et al. 2004) decreased the development of alcohol CPP (Lopez-Moreno et al. 2004). The development and enhancement of alcohol preference was also observed after chronic treatment with the CB_2_ agonist JWH 015 in stressed mice, but not in controls (Onaivi et al. 2008). Regarding WIN 55,212-2, it should be noted that higher doses of this drug injected systemically or into the ventral tegmental area provoked reduction of alcohol intake in mice (Linsenbardt and Boehm 2009). Increased AEA levels induced by inhibition of FAAH by URB597 evoked either a rise in alcohol consumption (Blednov et al. 2007; Hansson et al. 2007; Vinod et al. 2008a) or no effect in Marchigian Sardinian alcohol-preferring (msP) rats, and this treatment additionally had potent anxiolytic-like properties (Cippitelli et al. 2008). Interestingly, the increased levels of AEA after acute administration of the selective AEA reuptake inhibitor AM404 reduced the number of active lever responses in rats during alcohol self-administration. Additionally, AM404 did not affect the relapse induced by contextual cues associated with alcohol (Cippitelli et al. 2007). It should be noted that these effects were not mediated via CB_1_, CB_2_, or TRPV1 receptors but via other targets in the eCB system. On the whole, facilitation of brain eCB signaling seemingly contributes to alcohol consumption (Table 2).

**Table 2:** Representative eCB ligands in behavioral tests for alcohol use disorder

Drug	Dosage/route/treatment	Animals	Test	Result	References
eCB signaling activation					
Non-selective CB_1_/CB_2_ receptors agonists					
CP55,940	10, 30, 50 µg/kg; i.p.; acute	Wistar rat	Progressive ratio operant responding for beer	↑ motivation for beer	Gallate et al. 1999
	3, 10, 30 µg/kg; i.p.; acute	Sardinian alcohol-preferring (sP) rats	Voluntary alcohol intake (two-bottle choice)	↑ alcohol intake	Colombo et al. 2002
	10, 30, 50 µg/kg; i.p.; acute	C57BL6/J and DBA/2 mice	Voluntary alcohol intake (two-bottle choice)	↑ alcohol intake	Vinod et al. 2008b
WIN55,212-2	0.4, 2, 10 mg/kg; s.c.; 1 and 2 weeks after 7 days of deprivation	Wistar rats	Voluntary alcohol intake (two-bottle choice)	↑ alcohol intake	Lopez-Moreno et al. 2004
	0.4, 2, 10 mg/kg; s.c.; 1 and 2 weeks after 7 days of deprivation	Wistar rats	Conditioned place preference (CPP) procedure	↓ development of alcohol-conditioned place preference	Lopez-Moreno et al. 2004
	2 mg/kg; i.p.; 5 days during abstinence	Wistar rat	Alcohol-relapse model	↑ alcohol intake	Alen et al. 2009
	0.5, 1, 2 mg/kg; i.p.; acute	Sardinian alcohol-preferring (sP) rats	Voluntary alcohol intake (two-bottle choice)	↑ alcohol intake	Colombo et al. 2002
	0.5, 1, 2 mg/kg; i.p.; acute	C57BL6/J mice	Drinking in the Dark (DID) model	↑ alcohol intake (0.5 mg/kg) ↓ alcohol intake (1 and 2 mg/kg)	Linsenbardt and Boehm 2009
	0.25, 0.5, 2.5 µg/slide; microinjection into Ventral Tegmental Area	C57BL6/J mice	Drinking in the Dark (DID) model	↑ alcohol intake (0.25 and 0.5 µg/slide) ↓ alcohol intake (2.5 µg/slide)	Linsenbardt and Boehm 2009
Selective aea reuptake inhibitor					
AM404	0.4, 2, 10 mg/kg; i.p.; acute,	Wistar rats	Operant alcohol self-administration	↓ alcohol self-administration	Cippitelli et al. 2007
Faah inhibition					
URB597	0.4, 4 µg; microinjection into prefrontal cortex	Wistar rats	Operant alcohol self-administration	↑ alcohol self-administration	Hansson et al. 2007
	0.1, 0.3, 1 mg/kg; i.p.; acute	Wistar rats	Operant alcohol self-administration	ϕ	Cippitelli et al. 2008
			Cue-, footshock stress-and yohimbine-induced relapse	ϕ	
	0.1, 0.3, 1 mg/kg; i.p.; acute	Marchigian Sardinian alcohol-preferring (msP) rats	Voluntary alcohol intake (two-bottle choice)	ϕ	Cippitelli et al. 2008
	0.5 mg/kg; i.p.; acute	129/SvJ and C57BL6/J mice	Voluntary alcohol intake (two-bottle choice)	↑ alcohol intake	Blednov et al. 2007
	0.1 mg/kg; i.p.; acute	C57BL6/J mice	Voluntary alcohol intake (two-bottle choice)	↑ alcohol intake	Vinod et al. 2008a
eCB signaling inactivation					
CB1 selective antagonist					
SR141716A	0.3, 1, 3 mg/kg; i.p.; acute	Long-Evans rats	Operant alcohol self-administration	↓ alcohol self-administration	Freedland et al. 2001
	0.3, 1, 3 mg/kg; i.p.; acute	Wistar rats	Progressive ratio operant responding for beer	↓ break-points	Gallate et al. 1999
	0.3, 1, 3 mg/kg; i.p.; acute	Wistar rats	Operant alcohol self-administration	↓ alcohol self-administration	Rodriguez de Fonseca et al. 1999
	0.3, 1, 3 mg/kg; i.p.; acute	Wistar rats	Operant alcohol self-administration	↓ alcohol self-administration	Cippitelli et al. 2005
	0.3, 1, 3 mg/kg; i.p.; acute	Wistar rats	Cue-induced reinstatement to operant alcohol self-administration	↓ conditioned reinstatement of alcohol -seeking behavior	Cippitelli et al. 2005
	0.3, 1, 3 mg/kg; i.p.; acute	Wistar rats	Operant alcohol self-administration	↓ alcohol self-administration	Economidou et al. 2006
	0.3, 1, 3 mg/kg; i.p.; acute	Wistar rats	Cue-induced reinstatement to operant alcohol self-administration	↓ conditioned reinstatement of alcohol -seeking behavior	Economidou et al. 2006
	0.3, 1, 3 mg/kg; i.p.; acute	Wistar rats	Stress-induced reinstatement to operant alcohol self-administration	ϕ	Economidou et al. 2006
	1, 3 µg; microinjection into nucleus accumbens	Wistar rats	Operant alcohol self-administration	↓ alcohol self-administration	Caille et al. 2007
	0.3, 1, 3 mg/kg; i.p.; acute	Wistar rats	Operant alcohol self-administration	↓ alcohol self-administration	Cippitelli et al. 2008
	2.5, 5, 10 mg/kg; i.p.; acute	Sardinian alcohol-preferring (sP) rats	Voluntary alcohol intake (two-bottle choice)	↓ alcohol intake	Colombo et al. 1998
	0.3, 1, 3 mg/kg; i.p.; acute	Sardinian alcohol-preferring (sP) rats	Voluntary alcohol intake (two-bottle choice)	↓ alcohol intake	Serra et al. 2002
	0.03, 1, 3 mg/kg; i.p.; acute	Marchigian Sardinian alcohol-preferring (msP) rats	Operant alcohol self-administration	↓ alcohol self-administration ↓ conditioned reinstatement of alcohol -seeking behavior	Cippitelli et al. 2005
	1, 3, 10 mg/kg; i.p.; acute	Ethanol-preferring AA rats	Operant alcohol self-administration	↓ alcohol self-administration	Hansson et al. 2007
	3, 6 µg; microinjection into prefrontal cortex	Ethanol-preferring AA rats	Operant alcohol self-administration	↓ alcohol self-administration	Hansson et al. 2007
	3, 6 µg; microinjection into striatum	Ethanol-preferring AA rats	Operant alcohol self-administration	ϕ	Hansson et al. 2007
	2.5, 5, 10 mg/kg; i.p.; acute	Warsaw High-Preferring line of rats	Voluntary alcohol intake (two-bottle choice)	↓ alcohol intake	Dyr et al. 2008
	3 µg/g; i.p.; acute	C57BL6/J mice	Voluntary alcohol intake (two-bottle choice)	↓ alcohol intake in young mice	Wang et al. 2003
	1, 3, 5 mg/kg; i.p.; acute	C57BL6/J and DBA/2 mice	Voluntary alcohol intake (two-bottle choice)	↓ alcohol intake	Vinod et al. 2008b
SLV330	1, 3, 10 mg/kg; p.o., acute	Wistar rats	Operant alcohol self-administration	↓ alcohol self-administration	de Bruin et al. 2011
	1, 3, 10 mg/kg; p.o., acute	Wistar rats	Cue-induced reinstatement to operant alcohol self-administration	↓ conditioned reinstatement of alcohol -seeking behavior	de Bruin et al. 2011
SR147778	0.3, 1, 10 mg/kg/day; i.p.; during chronic pulmonary ethanol intoxication for 30 days	Wistar rats	Voluntary alcohol intake (two-bottle choice)-for at least 30 days	↑ alcohol intake (between 6 and 10 day)-dose 10 mg/kg	Lallemand and De Witte 2006
	0.3, 1, 10 mg/kg/day; i.p.; during chronic pulmonary ethanol intoxication for 30 days	Wistar rats	Voluntary alcohol intake (two-bottle choice)-after chronic alcoholization	↓ alcohol intake	Lallemand and De Witte 2006
JWH-073 (analogs 27 and 30)	10 mg/kg/day; i.p.; 3 days	NIH Swiss mice	Voluntary alcohol intake (two-bottle choice)	↓ alcohol intake	Vasiljevik et al. 2013
	10 mg/kg/day; i.p.; 3 days	NIH Swiss mice	Conditioned place preference (CPP) procedure	↓ development of alcohol-conditioned place preference	Vasiljevik et al. 2013
PF514273	1 and 5 mg/kg; i.p.; acute	DBA/2 J mice	Conditioned place preference (CPP) procedure	ϕ	Pina and Cunningham 2014
LH-21	0.03, 0.3, 3, 10 mg/kg; i.p.; acute	Wistar rats	Operant alcohol self-administration	↓ alcohol self-administration	Pavon et al. 2006

↑—increase, ↓—decrease, ϕ—none effect

In contrast with cannabinoid agonists, cannabinoid antagonists produce opposite effects, which mediate reduction in alcohol consumption. This conclusion is based on several observations using cannabinoid antagonists. SR141716A (rimonabant), a potent and selective CB_1_ antagonist, is one of the most well studied compounds and presents a link between cannabinoid receptors and alcohol consumption. The strong ability of this compound to inhibit or reduce the consumption of ethanol has been reported in alcohol-preferring rat strains, such as Sardinian alcohol-preferring (sP) rats (Colombo et al. 1998; Serra et al. 2002), Marchigian Sardinian alcohol-preferring (msP) rats (Cippitelli et al. 2005), alcohol-preferring AA rats (Hansson et al. 2007), and Warsaw High-Preferring rats (Dyr et al. 2008). Furthermore, in alcohol-preferring AA rats, a microinjection of SR141716A into the medial prefrontal cortex reduced alcohol self-administration, while microinjections into the dorsal striatum did not change the number of active lever responses for alcohol (Hansson et al. 2007). In another study, a reduction of alcohol self-administration was observed upon microinjection of SR141716A into the nucleus accumbens (Caille et al. 2007). Alcohol-preferring mice (C57BL/6J) with a genetic deletion of the functional copies of the CB_1_ gene demonstrated a decrease in voluntary alcohol intake comparable to pharmacological blockade using SR141716A administration in these mice (Vinod et al. 2008b; Wang et al. 2003). The reduced alcohol self-administration was also observed in rats after acute SR141716A administration (Cippitelli et al. 2005; Economidou et al. 2006; Freedland et al. 2001; Rodriguez de Fonseca et al. 1999) with reduced conditioned reinstatement of alcohol-seeking behavior (Cippitelli et al. 2005; Economidou et al. 2006). The recent study by Marinho et al. (2014) revealed that rimonabant abolished total and peripheral locomotion only at the dose of 10 mg/kg, while lower doses did not affect alcohol-induced hyperlocomotion (Marinho et al. 2014). Nevertheless, similar inhibitory activity and effectiveness was also observed after administration of other CB_1_ receptor antagonists: i) SVL330, which attenuated alcohol self-administration and reinstatement to alcohol-seeking behaviors in rats (de Bruin et al. 2011); and ii) SR147778, which reduced alcohol preference during cessation of chronic ethanol intoxication but not when co-administered with alcohol (Lallemand and De Witte 2006). This opposing behavior of SR147778 seemed to be similar to that observed for rimonabant. However, significant differences (e.g., induction of a shorter transient increase of alcohol intake) were noted. Additionally, Vasiljevik et al. (2013) reported that two of analogs of a monohydroxylated metabolite of the synthetic aminoalkylindole cannabinoid JHW-073 were shown to decrease alcohol self-administration and alcohol CPP, and, unlike rimonabant, they did not alter body weight during the treatment period (Vasiljevik et al. 2013). However, the ability to inhibit alcohol consumption was not reported for a novel CB_1_ receptor antagonist, PF514273, which did not reduce the acquisition or expression of alcohol-induced CPP (Pina and Cunningham 2014). On the other hand, LH-21 reduced alcohol self-administration (Pavon et al. 2006). Although the main interest is focused on the inhibitory activity of cannabinoids on alcohol abuse, their other potential therapeutic properties have been presented. The study conducted by Jeong’s group (2008) has indicated that a CB_1_ antagonist may slow down the development of alcohol fatty liver disease and thus prevent or delay its progression to more severe and irreversible forms (Jeong et al. 2008). Yang et al. (2014) proposed administration of CBD to prevent the liver damage caused by alcohol abuse. Additionally, eCB stimulation produced a neuroprotective effect on excitotoxicity induced by the cessation of chronic alcohol consumption (Rubio et al. 2011). Indeed, it has been reported that administration of HU-210, a CB_1_ receptor agonist, induced a significant protection against NMDA-induced cell death during either alcohol withdrawal or alcohol exposure, while chronic administration of a CB_1_ antagonist aggravated neuronal death induced by NMDA (Rubio et al. 2011) (Table 2). On the whole, these data point to the potential therapeutic application of CB antagonists in alcohol abuse.

#### Clinical Studies

As described above, preclinical studies provide a vast body of evidence suggesting the potential use of the CB_1_ antagonists in the treatment of alcohol abuse. Unfortunately, the response of alcoholic patients to rimonabant treatment was not so beneficial. Soyka et al. (2008) examined the effect of rimonabant (20 mg per day for 12 weeks) on the rate of relapse among recently detoxified alcoholic individuals and found that rimonabant was ineffective in preventing relapse (Soyka et al. 2008). In the line with this trial, another study using rimonabant (20 mg per day for 2 weeks) in non-treatment-seeking heavy alcohol drinkers demonstrated no effect of this drug on alcohol consumption (George et al. 2010). These two groups failed to find significant differences between rimonabant and placebo in the experiments determining drug impact either on the time to first drink and relapse to heavy drinking or on overall consumption. Several aspects of this lack of effectiveness were considered, such as incorrect administration and small numbers of clinical subjects. However, in consequence, considering an analogical study with naltrexone used at higher doses of 50–150 mg/day, which significantly decreased the consumption of alcohol (Weerts et al. 2008), and the fact that both cannabinoids and naltrexone are inhibitors of the mesolimbic dopamine reward system, it has been suggested that rimonabant used at a dose 20 mg/day only partially blocked the CB_1_ receptor. Therefore, a suggestion was put forth that a significant decrease in the motivation to drink alcohol can occur only as a result of a complete blockade of the signaling associated with the reward system (Cippitelli et al. 2005).

In human clinical studies, marijuana was found to exert some beneficial effects. A prospective study of heavy drinkers revealed that administration of cannabis (marijuana) reduced alcohol consumption faster than in non-cannabis users (Metrik et al. 2011). Marijuana and some cannabinoid ligands are known to have influence not only on the consumption of alcohol but also on several effects related with alcohol. Marijuana has been shown to decrease the risk of head neck cancer among moderate tobacco smokers and light alcohol drinkers (Liang et al. 2009).

## Adverse Effects Induced by a Combination of Cannabinoid Ligands and Alcohol

Alcohol abuse is one of the main causes of death and disability in developed countries. Together with cannabinoids, it is considered to be one of the most popular drugs among recreational users. There are explicit similarities between acute effects of ethanol and cannabinoids (Hungund and Basavarajappa 2000). At low doses, both stimulate activity and euphoria, whereas at high doses they produce sedation, and lack of motor coordination (Hungund and Basavarajappa 2000), even if the levels of the consumed doses at which these effects take place differ. Several behavioral and pharmacological effects induced by alcohol have been shown to be the same as those produced by THC. An intake of these substances induces a disruption of spatial learning (Cha et al. 2006), motor dysfunction (Dar 2014), and analgesia (Chiou et al. 2013).

Alcohol and cannabinoids consumed separately lead to several undesirable side effects. Alcohol is found to produce various effects depending on the dosage and duration of its consumption. Low doses of alcohol elicit appetitive gustatory responses that improve the taste of beverages, such as beer or wine (Lemon et al. 2004). In addition, small amounts of alcohol improve mood and generally reduce anxiety. On the contrary, long-term drinking of higher doses of alcohol results in memory impairment, sedation, motor incoordination, confusion, hypothermia, and sometimes vomiting (Gordon and Devinsky 2001; White 2003; Zoethout et al. 2011). Some individuals who are heavy chronic drinkers are at risk of coma and death caused mainly by respiratory depression (Vonghia et al. 2008). Additionally, alcohol is a well known substance most strongly associated with aggression (Hoaken and Stewart 2003), liver diseases, as well as cardiomyopathy (Klatsky 2007).

Similar to alcohol, both endogenous cannabinoids and synthetic CB_1_ receptor agonists are well known to impair learning skills and memory (Croft et al. 2001). Moreover, acute intoxication with cannabis leads to transient episodes of confusion, depersonalization, paranoid delusions, hallucinations, blunted affect, anxiety, and agitation (Fernandez-Espejo et al. 2009). In addition to these variety of effects, the consumption of cannabis affects psychomotor activity (McLaughlin et al. 2000).

Although some have found no acute addictive effects of co-administered cannabinoids and alcohol, it is postulated that such a combination may enhance some of their inherent adverse effects.

### Preclinical Studies

There are several lines of evidence confirming a synergic action of Δ^9^-THC and alcohol, which increase the risk of their co-administration. One study reporting side effects resulting from concomitant alcohol and marijuana use was presented by Ciccocioppo et al. (2002), who demonstrated that memory disturbances might be aggravated by a co-administration of cannabinoids and alcohol. In fact, a significant impairment of object recognition was observed in msP rats injected with Δ^9^-THC (at a dose of 10 mg/kg, i.p.) after alcohol intake, while lower doses did not change this parameter (Ciccocioppo et al. 2002). It was also observed that intracerebellar injection of Δ^9^-THC antagonized alcohol-induced ataxia following alcohol consumption in mice (Dar 2014).

### Clinical Studies

It has been shown that both alcohol and marijuana use resulted in significant changes in brain structures as well as abnormal brain functioning, which were much more serious than those produced by each substance separately. These changes mostly affect the hippocampus, which when damaged, may lead to deficits in verbal and visual memory, working memory, visuospatial functioning, gait/balance, reasoning, response perseveration, and processing speed (Aasly et al. 1993; Gillet et al. 2001; Lisdahl et al. 2013; Medina et al. 2007; Tapert et al. 2002). Recent studies by Winward et al. (2014) have shown that concomitant use of marijuana and alcohol during adolescence evoked decrements in cognitive functioning and poor performance in specific cognitive domains. Additionally, differences in verbal recall as well as in cognitive flexibility were observed even after a month of abstinence from concomitant use of marijuana and alcohol in comparison with the control group (Winward et al. 2014). Surprisingly, considering the structural changes of the hippocampus, it was reported that individuals using a combination of these two substances did not differ from non-substance using controls in hippocampal asymmetry or volume, which was explained by the specific mechanism of marijuana alone (Medina et al. 2007). Such buffering action of cannabinoids on the negative effects of alcohol on the brain was also shown by Mahmood et al. (2010) who indicated no relation between visual learning or memory performance and alcohol hangover/withdrawal in cannabis-using adolescents (Mahmood et al. 2010). Additionally, cumulative acute effects of Δ^9^-THC or CBD and alcohol in perceptual and motor function have been found (Belgrave et al. 1979; Bramness et al. 2010; Chait and Perry 1994; Consroe et al. 1979). The combination of these drugs is also well known to increase reaction time and the number of incorrect responses to emergencies (Bramness et al. 2010). Several studies have found additive or multiplicative effects of marijuana and alcohol on causing road accidents (Bramness et al. 2010; Ramaekers et al. 2000). Dubois et al. (2014) reported that drivers positive for both substances had a greater likelihood of making errors than drivers positive for either alcohol or cannabis alone (Dubois et al. 2014). This was observed in parallel with the study by Bramness et al. (2010), who revealed that simultaneous application of both substances impaired driving ability, thus markedly increasing the risk of being judged impaired (Bramness et al. 2010). Additionally, Ronen et al. (2010) reported that during driving tests, either heart rate remained higher or greater sensations of fatigue and sleepiness over time were observed after administration of the combination of these two drugs (Ronen et al. 2010). However, it has been suggested that driving performance affected by both alcohol and THC consumption depends on the doses of both substances as well as on the drug-use history of the individual, and thus, this issue still remains inconclusive. It is worthwhile to note that a combination of cannabinoids and cannabis-like substances with alcohol may induce aggressive behavior. This assumption is based on the findings of Pennings et al. (2002) who reported that aggressive behavior may be magnified when alcohol and other illicit drugs (such as cocaine) are combined (Pennings et al. 2002). On the other hand, Easton et al. (2007), while examining the differences between an alcohol alone group and an alcohol + drug (cocaine or marijuana) group, noticed that the participants who combined alcohol with another drug had problems with anger management (increases in angry feelings and anger expression such as slamming doors). However, this did not result in physical violence against their partners or other people (Easton et al. 2007). Indeed, a recent study by Korcha et al. (2014) confirmed these assumptions by showing that in subjects who reported using a combination of alcohol and drugs (marijuana), men were seven times more likely and women were four times more likely to report a violence-related injury compared with those reporting no use of either alcohol or drugs prior to injury (Korcha et al. 2014). A recently published article by Kelly et al. (2015) has suggested that mid-adolescent subjects (14–15 years old) characterized as polydrug users (mainly alcohol and cannabis users) are at an elevated risk of school non-completion (Kelly et al. 2015).

Interestingly, while cannabinoid ligands may serve as a useful tool for reduction of alcohol withdrawal symptoms, it has been revealed that cannabis abstinence leads to significant increases in alcohol consumption among those with a previous alcohol dependence diagnosis or those with low alcohol consumption at baseline (Allsop et al. 2014; Peters and Hughes 2010). This was in agreement with the previous findings of Midanik et al. (2007) who reported that concurrent alcohol and marijuana users were not only characterized by a greater alcohol dependence but also experienced greater social consequences and depression (Midanik et al. 2007). Furthermore, Osilla et al. (2014) have shown that people using both marijuana and alcohol reported increased alcohol consumption, both with regards to frequency and quantity, as well as greater alcohol-related consequences and prescription drug misuse (Osilla et al. 2014).

## Conclusions

In this review, we focused on beneficial and adverse effects resulting from a combination of well-known and widely used psychoactive stimulants: alcohol and cannabinoids.

According to presented data, the magnitude of potential side effects induced by these two substances seems to dominate,. There are several reports demonstrating that a co-administration of cannabinoid receptor antagonists and alcohol may be a highly effective therapy for alcohol abuse. However, this effect only applies to animal studies because rimonabant, for example, failed to be active in human studies. While its administration to animals resulted in a significant reduction in alcohol consumption and an attenuation of alcohol-induced hyperlocomotion, the application of rimonabant to patients (either detoxified or non-treated heavy alcohol drinkers) revealed to have no effect on drinking. Importantly, rimonabant has been withdrawn from the market due to potentially serious side effects. Therefore, even if its intake were proved to be beneficial in terms of reduction of alcohol consumption or inhibition of alcohol withdrawal symptoms, this drug would require supervised use. Interestingly, the use of cannabinoid receptors antagonists in animals is unclear. It has been shown that different antagonists exert different, and sometimes opposite, effects (see: AM630, PF514273, and LH-21). Additionally, drug distribution and affected brain area were found to be of a great importance. Moreover, with regard to cannabinoid receptor agonists, data presented for clinical and preclinical studies indicated a surprising difference in action. A great example is marijuana, which intensifies the desire to drink alcohol in animals but reduces alcohol consumption in human cannabis users. This is consistent with the overall sentiment based on various preclinical studies in rodents that the pharmacological blockade or genetic ablation of CB_1_ receptors decreased operant self-administration of alcohol and its voluntary consumption. Furthermore, the activation of CB_1_ receptors facilitates alcohol consumption while antagonism of CB_1_ receptor reduces the motivational properties of alcohol.

In light of this highly inconsistent information and the fact that results obtained in animals do not necessary translate into human studies, it is very difficult to unanimously say that cannabinoid drugs may be useful in human therapy. This, however, may change thanks to novel pharmacological strategies involving the use of CB_2_ receptor agonists. Although, their role in the treatment of alcohol dependence is still under investigation and a development of a drug that possesses the same activity both in animals and human is greatly needed, CB_2_ agonists (particularly highly selective ones) may be much more useful than CB_1_ ligands due to the lack of psychotropic effects mediated by cannabinoid 1 receptors, despite the fact that CB_1_ antagonism appeared to be favorable for the treatment of alcohol dependence.

## Abbreviations

AEA: Anandamide
2-AG: 2-Arachidonoylglycerol
eCBs: Endocannabinoids
FAAH: Fatty acid amide hydrolase
